# Coastal Futures: Our philosophy, our impact

**DOI:** 10.1017/cft.2026.10047

**Published:** 2026-07-31

**Authors:** Thomas Spencer

**Affiliations:** Geography, Cambridge University, UK

In a position piece published in Volume 1, July 2023, the Senior Editors of *Cambridge Prisms: Coastal Futures* argued that understanding the future of coastal environments requires a much wider and more sophisticated framing than that of the simple interaction between increasing hazards (typically related to rising sea levels) and the increasing exposure and vulnerability of rapidly growing coastal populations. This inadequate, generalised, often global-scale, view needs to be extended into debates that stress: ‘the importance of place-and people-based biophysical systems and social contexts; the need for technical innovation in restoring and rehabilitating degraded coastal environments; and the identification of just and equitable adaptive response pathways towards better, more robust coastal futures’. The Editors identified the ‘coupled dance between human decisions and coastal environmental change’, in essence ‘a coupled social – ecological – physical system’ (Spencer et al., 2023). Thus, the journal has aimed to define and inhabit a particular ‘thought space’, with submissions recording interdisciplinary (as opposed to simply multidisciplinary) research being especially encouraged.

So the journal set out its stall, provided focus to prospective authors through an online Topic Map (Cambridge University Press & Assessment, 2026) and, with the considerable and sustained help of the Senior Editors and Editorial Board (where we have worked hard to assemble a diverse and inclusive community of editors), has both proactively sought submissions and advertised more generally. In a not-so-uncrowded field for coastal and environmental journals, how have we been doing?

An assessment from May 2026, where submissions were mapped onto the Topic Map classes, shows a wide spread of papers, the top two categories being ‘climate change’ and ‘conservation and restoration’ but with good representation of ‘geomorphology’, ‘society and culture’, ‘coastal development’, coastal monitoring’ and ‘governance’. Just as the Editors had hoped. We have published a Themed Collection on ‘Transdisciplinary Science for Near-Future Habitable Coasts’ and continue to add to the Collections on ‘Extreme Events at the Coast’ and ‘System Impacts of Nature Based Solutions for Coastal and Water Management’ (joint with *Cambridge Prisms: Water*). There are several more Collections at the developmental stage. The broad themes mentioned above are not the only ones published in the journal, however, and have indeed been supported by more focused papers on carbon sequestration and coastal engineering. The purpose of the journal is first and foremost the publication of first-rate coastal papers, including in specialist fields.

The geographical spread of submissions is broad (Figure 1) but unsurprising; over 30% of authors are from Europe, ca. 20% from the United States and 12% from Australia, but only 4% from the whole of Africa (and most of these submissions from South Africa). Ninety-seven percent of authors are from high- to upper-middle-income countries (HIC/UMIC, using World Bank definitions). We are of course concerned with the dangers of publishing ‘parachute science’, whereby international scientists from high-income countries undertake field studies in lower-income countries (LMIC/LIC), often using local resources and mining indigenous knowledge, but then writing up their research back home without any further effective communication and engagement with the host nation. We now ask, where appropriate, for authors to provide an inclusivity statement that documents the involvement of local communities throughout the planning and implementation of the research reported in the submission. And we wish to go further and support the true coproduction of research between local communities and academic researchers. We are encouraging this practice by encouraging the submission of perspective articles that capture different voices on real local research needs and perceived coastal futures and by collaborative submissions between scientists in HIC/UMIC and LMIC/LIC countries. We have some such collaborations published and more in the system – but we would like to see more.

**Figure 1.**
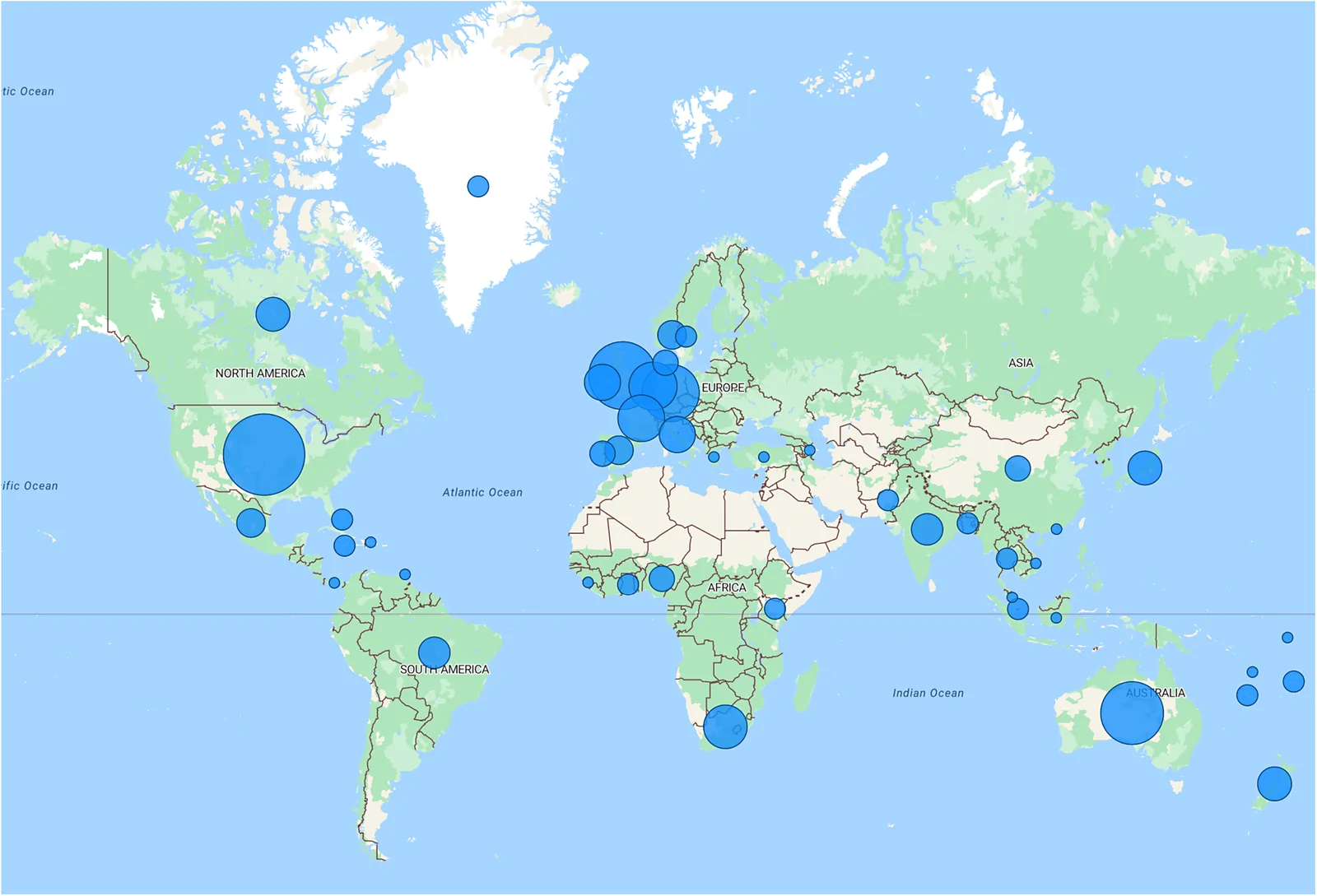
Geographical locations of all journal authors and co-authors, all time periods (circle sizes are indicative only). Figure 1. long description.

Finally, some numbers. As I hope the above makes clear, our prime focus at *Cambridge Prisms: Coastal Futures* revolves around attracting authors who share our vision of publishing high-quality research with a strong focus on the potentialities and challenges of interdisciplinary science. But for the waverers, the numbers are truly impressive for a young journal. Overall, the journal has recorded over 300,000 downloads and 670 citations, with an Impact Factor that sits at 8.5, placing us in Q1 for both Environmental Sciences (47/395, 88.2 JIF Percentile) and Marine & Freshwater Biology (3/124, 98.0 JIF Percentile).

Come and join us.

## Data Availability

Not applicable.

## Long descriptions

### Figure 1. Long description

A world map displays the global distribution of authors using blue circles. The highest concentration and largest circles are located in Western Europe and North America, particularly the United States. In Europe, large overlapping circles cover the United Kingdom, France, and Germany. In North America, a very large circle is centered over the central United States, with smaller circles in Canada and Mexico.

Moving East, Asia shows moderate activity with circles in India, China, Japan, and Southeast Asia. Australia features one large circle in its southeastern region and several smaller ones in the surrounding islands. South America has a notable concentration in Brazil. Africa shows sparse distribution with small circles primarily in the West, East, and a larger one in South Africa. A single small circle is placed on Greenland. The circles are overlaid on a standard map showing continental outlines and labels for North America, South America, Africa, Europe, Asia, and Australia.

Navigate back to Figure 1..
