# Supplementary material for: Coastal Futures: Our philosophy, our impact
Source: Camb Prism Coast Futur. 2026 Jul 31;4:e23. doi: 10.1017/cft.2026.10047 (PMC13748879; doi:10.1017/cft.2026.10047)
Supplement: Peer review history

# Editor report: Recommendation: Coastal Futures: Our philosophy, our impact — R0/PR2

**DOI:** 10.1017/cft.2026.10047.pr2

**Affiliations:** Cambridge University Press & Assessment, United Kingdom of Great Britain and Northern Ireland

No accompanying comment.

# Editor report: Decision: Coastal Futures: Our philosophy, our impact — R0/PR3

**DOI:** 10.1017/cft.2026.10047.pr3

**Affiliations:** Cambridge University Press & Assessment, United Kingdom of Great Britain and Northern Ireland

No accompanying comment.
